# Insights into Growth of a Photoactive Layer Based on Perylene Diimide Bearing Alkoxysilane Groups

**DOI:** 10.1021/acs.langmuir.5c03319

**Published:** 2025-09-22

**Authors:** Karolina Socha, Maciej Krzywiecki, Patryk Mroczko, Dawid Nastula, Karol Erfurt, Radosław Motyka, Agata Blacha-Grzechnik

**Affiliations:** 1 Faculty of Chemistry, Silesian University of Technology, Strzody 9, Gliwice 44-100, Poland; 2 Institute of Physics−CSE, Silesian University of Technology, Konarskiego 22B, Gliwice 44-100, Poland; 3 Centre of Polymer and Carbon Materials of the Polish Academy of Sciences, 34 M. Curie-Sklodowskiej St., Zabrze 41-819, Poland; 4 Centre for Organic and Nanohybrid Electronics, 49569 Silesian University of Technology, 22B Konarskiego St., Gliwice 44-100, Poland

## Abstract

The chemical grafting of organic photosensitizers onto solid surfaces has gained increasing attention lately as an effective strategy for the formation of stable, functional layers, with promising applications in areas such as antimicrobial coatings and optoelectronic devices. In this work, a perylene diimide (PDI) derivative bearing alkoxysilane groups, APTES-PDI-APTES, was synthesized and chemically grafted onto a glass surface. The presence of two alkoxysilanes enabled competing processes to occur, i.e., condensation reactions, which led to the formation of complex multilayer structures, and covalent bonding to the glass surface. The influence of deposition process parameters, such as solvent polarity and immersion time, on the chemical structure and the morphology of the resulting layer was studied using X-ray photoelectron spectroscopy, Raman spectroscopy, and atomic force microscopy. The singlet oxygen photogeneration was tested using an indirect detection method, proving that after immobilization, APTES-PDI-APTES retains its photosensitizing properties. These findings highlight the influence of the process parameters on the development of the PDI-containing multilayer and its potential application as a heterogeneous source of singlet oxygen.

## Graphical abstract

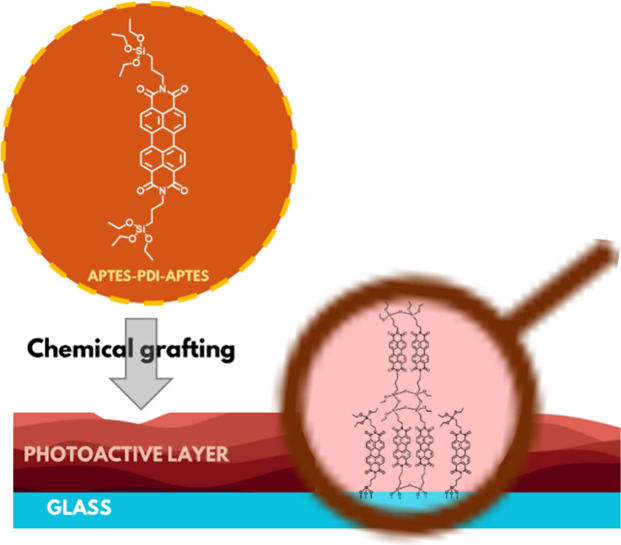

## 1 Introduction

Self-assembled monolayers (SAMs) have been of research interest since their discovery by Nuzzo and Allara in the 1980s.^1^ They have been considered as a promising material for application in various fields, e.g., electronics,^2–4^ catalysis,^5–10^ biosensors,^11,12^ medical diagnostics,^13–16^ photovoltaics,^17–20^ or as antimicrobial coatings.^21–24^ Modifying organic compounds’ structure by introduction of surface-anchoring groups enables the tailoring of the physicochemical properties of solid surfaces depending on specific needs. Among various functional groups that undergo self-assembly on a surface, thiol- and alkoxysilane-based SAMs have been the most widely studied.^25,26^ Historically, the initial research related to SAMs focused mainly on compounds containing thiol groups due to their ability to form well-ordered and stable layers on noble metal surfaces.^27–29^ Next, the growing interest in the chemical grafting of alkoxysilanes opened up new possibilities in the surface functionalization, particularly considering silicon oxide,^30,31^ titanium oxide,^32^ and zinc oxide.^33,34^ Compounds containing a silane group in the structure offer covalent chemical bonding and stability on a wider range of substrates.^35^ This makes them particularly attractive for emerging hybrid systems in electronics, sensors, and other advanced applications.^36,37^

With the rising prevalence of viral and bacterial infections, the development of surfaces with antimicrobial properties has become increasingly important.^38–40^ Recently, the deposition of layers of organic photosensitizers (PSs) has emerged as an effective strategy to prevent the accumulation of microorganisms on inanimate surfaces. Such photoactive layers, capable of generating singlet oxygen and other reactive oxygen species, are versatile and highly effective against various bacteria, viruses, and fungi.^41–48^ The important feature of such PS-based coatings is long-term stability, i.e., no leakage of the photosensitizer into the environment and photostability, that can be provided by covalent attachment of PSs to the surface. Such photoactive antimicrobial coatings formed by chemical grafting have been already reported on various substrates, including glass,^49,50^ lignocellulosic fibers,^51^ and cotton fabrics.^52^

Perylene diimides (PDIs), due to their outstanding electron transport properties, were initially considered for application in organic photovoltaic devices.^53,54^ Lately, their ability to generate singlet oxygen has gained increasing attention, particularly for anticancer photodynamic therapy (PDT) and photodynamic antimicrobial therapy (PACT).^55–57^ PDI derivatives typically exhibit a singlet oxygen quantum yield, Φ_Δ_, of around 0.2.^58,59^ However, appropriate substitution in the *bay* position, e.g., with donor groups, allows to increase the Φ_Δ_ value even up to 0.7–0.9.^57,60,61^ This, together with the PDI’s strong absorbance in UV and vis regions, offers great potential for the application of PDI-based photosensitizers.

In this work, we explored the possibility of the formation of a PDI-based photoactive multilayer covalently attached to the glass surface. With this aim, a perylene diimide derivative bearing two alkoxysilane groups substituted in imide positions (APTES-PDI-APTES) was synthesized. The alkoxysilane group primarily allows for the chemisorption on the glass surface, yielding a self-assembled monolayer.^62,63^ On the other hand, −Si­(OR)_3_ groups undergo a condensation reaction to form porous materials.^64–68^ Hence, during the chemical grafting of APTES-PDI-APTES, two competitive processes should occur (Figure 1), yielding multilayer structures covalently attached to the solid surface. The influence of the deposition process conditions, i.e., polarity of the solvent and immersion time, was studied. The resulting layers were characterized by Raman and X-ray photoelectron (XPS) spectroscopies and atomic force microscopy (AFM). The photosensitizing properties of APTES-PDI-APTES, in the solution phase and in the form of a layer, were investigated by the indirect method, applying UV–vis spectroscopy and a specific ^1^O_2_ trap.

**Figure 1:**
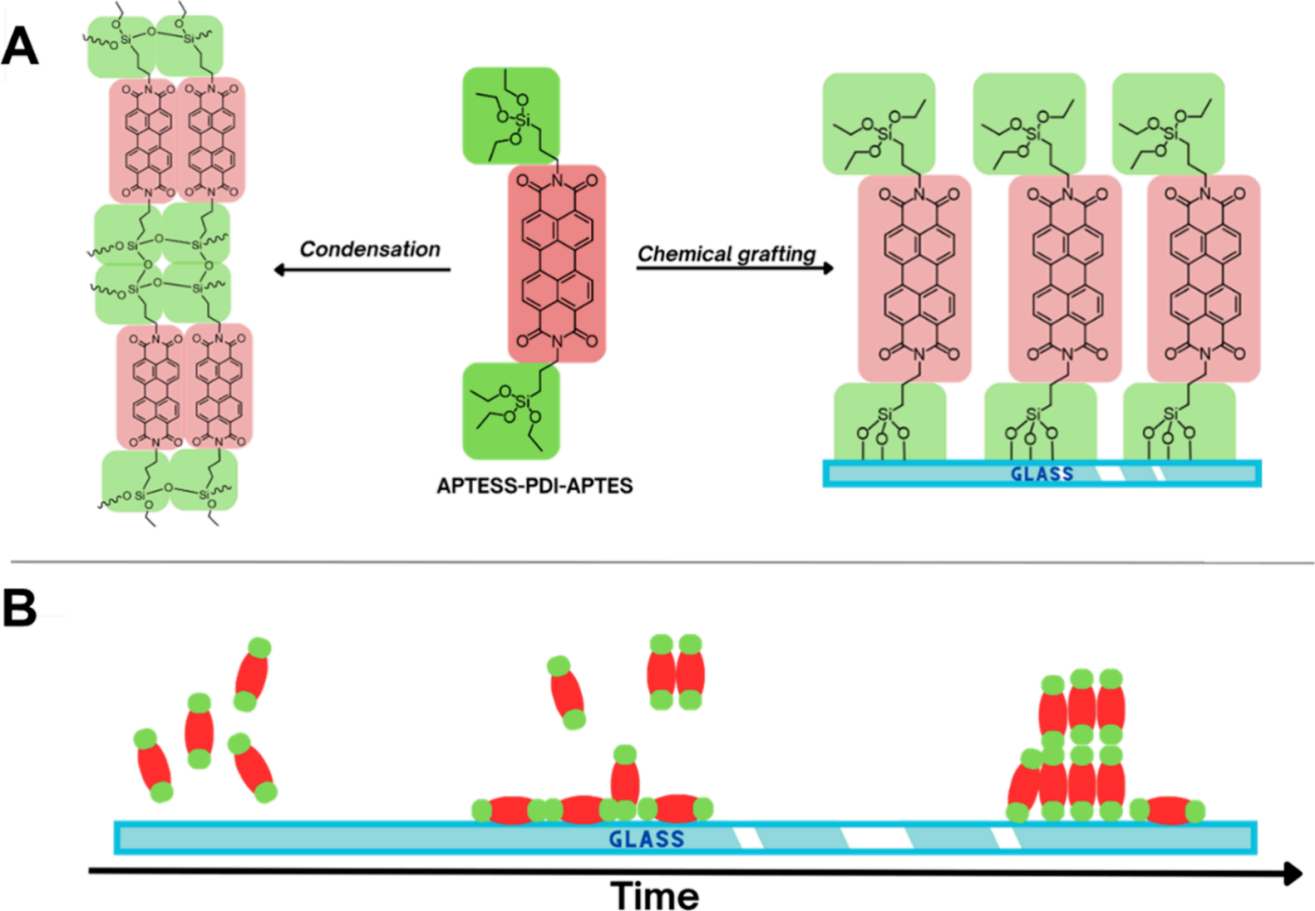
(A) Possible reaction routes of bonding of perylene diimide bearing alkoxysilane groups. (B) Schematic representation of APTES-PDI-APTES monolayer growth.

## 2 Experimental Section

### 2.1 Materials

3,4,9,10-Perylenetetracarboxylic dianhydride (Fluorochem, purity 98%) and (3-aminopropyl)­triethoxysilane (Acros Organics, purity 99%) were used for the synthesis of APTES-PDI-APTES (Scheme S1). The APTES-PDI-APTES compound was synthesized according to a modified literature procedure.^65^ Details of the preparation, purification, and characterization (^1^H NMR, ^13^C NMR, or HRMS spectra) of the compound in question are described in the Supporting Information. The 1,3-diphenylisobenzofuran (DPBF, purity >97%) used for singlet oxygen generation studies was purchased from Acros Organics.

The organic solvents *n*-hexane (99%), acetone (95%), and chloroform (99.8%) were purchased from Acros Organics (Geel, Belgium). Dichloromethane (HPLC grade) was purchased from Sigma-Aldrich. *N,N*-Dimethylformamide (DMF), dimethyl sulfoxide (DMSO), and toluene (extra dry, molecular sieve, AcroSeal) were purchased from Thermo Scientific. BOROFLOAT 33 borosilicate glass (1 cm × 1 cm, Präzisions Glas & Optik GmbH) was used as a substrate.

### 2.2 Characterization of APTES-PDI-APTES

The chemical structure of APTES-PDI-APTES was confirmed using infrared spectroscopy with attenuated total reflectance mode (ATR-IR), nuclear magnetic resonance spectroscopy (NMR), and mass spectrometry (MS). The ATR-IR spectrum was collected using a Spectrum Two N FT-NIR (Waltham, MA, USA) spectrometer, PerkinElmer. ^1^H NMR spectra were acquired on a Varian 400 spectrometer at an operating frequency of 400 MHz by using tetramethylsilane (TMS) as the resonance shift standard. ^13^C NMR spectra were recorded on a Varian 400 instrument at 100 MHz, using solvent resonance as the internal standard. All chemical shifts (δ) are reported in parts per million and coupling constants (*J*) in Hz. High-resolution mass spectrometry (HRMS) was performed using a Waters Xevo G2 Q-TOF mass spectrometer equipped with an ESI source operating in the positive ion mode. The accurate masses and compositions of molecular ions were calculated using Mass Lynx software incorporated within the instrument.

An Avantes AvaLight-DHc spectrophotometer and a Hewlett-Packard 8452A UV–vis spectrometer were used for UV–vis absorption and singlet oxygen measurements, respectively. In both cases, a dichloromethane solution of APTES-PDI-APTES was used. The measurements were carried out in a quartz cuvette (10 × 4 mm, Hellma Analytics). Singlet oxygen generation was studied using 1,3-diphenylisobenzofuran (DPBF, Acros Organics, purity >97%) as a chemical trap, prepared as a 0.05 mM solution in dichloromethane. The concentration of the test compound was 0.03 mM in the cuvette. Illumination was provided by a 532 nm diode laser (Oxxius, model LCX-532L-150-CSB-PPA) with a maximum output power of 150 mW, operating at 20 mW during the experiment. The quantum yield of singlet oxygen was determined using the so-called relative method^69^ with methylene blue (MB) as a reference.^70^

Fluorescence measurements were carried out using a fluoroSENS Pro11. The photoluminescence (PL) excitation wavelength (487 nm) was selected from the λ_max_ absorption using a UV–vis photospectrometer (UV3101PC).

### 2.3 Formation of APTES-PDI-APTES@glass

The glass slides were pretreated following procedures described in the literature.^71^ Initially, glass slides with an area of 1 cm^2^ were ultrasonicated with acetone for 15 min, followed by ultrasonication in deionized water for 15 min. The glass slides were then immersed in 1 M NaOH for 1 h. They were then sonicated again in deionized water for 15 min, followed by treatment with concentrated HCl for 1 h. Such pretreatment procedure aims to remove organic and inorganic contaminants and to activate the surface by increasing the number of hydroxyl groups, facilitating the grafting process.^72,73^

A grafting solution was prepared by dissolving 4 mg (0.005 mmol) of APTES-PDI-APTES in 5 mL of dry toluene, DMF, or DMSO in a Teflon beaker. The pretreated glass slides were thoroughly dried and immersed in the prepared solution. The atmosphere above the beaker was purged with argon. The top of the beaker was tightly wrapped with aluminum foil to limit light exposure and solvent evaporation. After a set time, the slide was taken out of the vessel and copiously washed with chloroform to remove any weakly attached species.

### 2.4 Characterization of APTES-PDI-APTES@glass

#### 2.4.1 Chemical Structure

The chemical structure of deposited layers was investigated by Raman and X-ray photoelectron spectroscopies. Raman spectra of APTES-PDI-APTES@glass were collected by a Renishaw inVia Raman microscope (Renishaw, Inc., New Mills, UK) with a 514 nm diode excitation laser and 2400 lines/mm grating. The recorded spectra were subjected to smoothing and baseline subtraction by applying Renishaw software.

X-ray photoelectron spectroscopy (XPS) analysis was performed using an AXIS Supra+ instrument (Kratos Analytical) equipped with a monochromatic Al Kα X-ray source (*h*ν = 1486.6 eV, operating at 10 mA, 15 kV). The system base pressure was equal to *p*_b_ = 2.3 × 10^–9^ Torr. The pass energy was set to 160 eV (scanning step 0.9 eV) or 20 eV (scanning step 0.05 eV) for survey spectra and for high-resolution spectra, respectively. For the charging effect compensation, the Kratos charge neutralization dual-beam system was used. The spectra were acquired at normal to the surface, unless stated otherwise. In order to investigate the homogeneity of the layer, the XPS spectra were acquired at least at three different points. The binding energy scale was calibrated with respect to the C–C component of C 1s spectra (284.8 eV). The acquired spectra were analyzed using CasaXPS software and embedded algorithms. The components of the high-resolution spectra were presented with Gaussian (70%) and Lorentzian (30%) lines, while the background was with Shirley’s function.

#### 2.4.2 Morphology

The AFM experiments were conducted using a Park Systems XE-70 microscope working in noncontact mode (NC-AFM). BudgetSensors TAP300DLC tips (resonant frequency 300 kHz, diamond-like coating) were utilized in order to reduce the possible humidity impact on the scanning process. Postprocessing of the images was done using Gwyddion software providing scanner-induced and inclination correction tools.^74–77^ Corrected surface images were quantitatively analyzed by Gwyddion built-in statistical analysis tools.^78^ For the statistical analysis of the surface topography (particularly grain size distribution), the watershed algorithm and grain analysis tools^79^ were applied.

#### 2.4.3 Photosensitizing Properties

The photogeneration of singlet oxygen from the layers was investigated under conditions analogous to those for the APTES-PDI-APTES solution. In this case, a glass plate coated with the tested layer was placed in a quartz cuvette and positioned perpendicular to the laser beam and parallel to the spectrophotometer detection path while ensuring that the beam path remained undisturbed. A 532 nm diode laser (Oxxius, LCX-532L-150-CSB-PPA model, 150 mW maximum power, 50 mW applied) served as the illumination source. UV–vis spectra of DPBF in methanol were recorded with a Hewlett-Packard 8452A UV–vis spectrometer.

## 3 Results and Discussion

### 3.1 Optical and Photosensitizing Properties of APTES-PDI-APTES

Figure 2A presents the normalized absorption and fluorescence spectra of APTES-PDI-APTES solution in DCM. The absorption and fluorescence spectra exhibit three bands located within 420–550 and 520–650 nm ranges, respectively. Such spectra are characteristic of the alkyl-substituted perylene diimide derivatives,^80,81^ indicating that the influence of −Si­(OR)_3_ on the optical properties is minimal. APTES-PDI-APTES exhibits a fluorescence quantum yield of 70% in DCM (Table 1).

**Figure 2:**
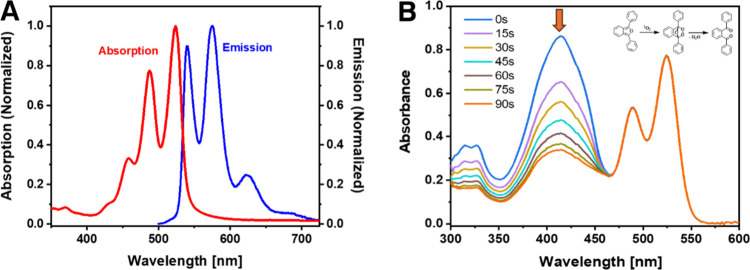
(A) Normalized absorption and fluorescence spectra of APTES-PDI-APTES in the DCM. (B) Set of UV–vis spectra of DPBF in DCM recorded during illumination of APTES-PDI-APTES solution with a 532 nm laser.

**Table 1:** Optical and Photosensitizing Parameters of APTES-PDI-APTES in DCM

λ_abs_ (nm)	ε_abs_ (1/M^–1^ cm^–1^)	λ_em_ (nm)	Φ_em_	Φ_Δ_ ^a^
458	7620	541	0.70	0.20
487	17,777	575		
524	23,459	623		

a Reference MB in DCM (Φ_Δ_ = 0.57).

The singlet oxygen generation was investigated by an indirect method using a DPBF chemical trap. DPBF exhibits a maximum absorption at 412 nm, which diminishes in the presence of APTES-PDI-APTES during 532 nm laser illumination (Figure 2B). This can be attributed to the reaction of DPBF with singlet oxygen generated by PDI, leading to the formation of 1,2-dibenzoylbenzene.^82^ In contrast, a control experiment conducted under identical conditions, but without the presence of a PDI photosensitizer, showed no significant changes in DPBF’s absorption during irradiation with the green laser. No decrease in the intensity of the absorption band of APTES-PDI-APTES was observed during illumination, indicating that the compound is photostable. The singlet oxygen generation quantum yield is equal to 20% in dichloromethane (Table 1), which corresponds to the values typically observed to PDIs only with alkyl chains in the imide positions.^59^

### 3.2 Formation and Characterization of APTES-PDI-APTES@glass

The organic layers were formed by using a straightforward procedure of chemical grafting. The pretreated glass slides were dipped into a solution containing the APTES-PDI-APTES compound. In this approach, two competitive processes involving trialkoxysilane groups can occur (Figure 1A). The hydrolyzed −SiOCH_3_ groups undergo a condensation reaction and can further form the covalent bond with the glass surface atoms or with each other. The latter serves as a basis for silica formation via the sol–gel method.^83^ Variation of the APTES-PDI-APTES parameters, specifically the immersion time and solvent type (Table 2), allowed for an understanding of the layer formation mechanism.

**Table 2:** Labels of the APTES-PDI-APTES@glass Layers Deposited under Various Process Conditions

sample name	solvent	immersion time
APTES-PDI-APTES@glass (DMF, 24h)	dimethylformamide (DMF)	24 h
APTES-PDI-APTES@glass (DMSO, 24h)	dimethyl sulfoxide (DMSO)	24 h
APTES-PDI-APTES@glass (tol, 3h)	toluene	3 h
APTES-PDI-APTES@glass (tol, 6h)		6 h
APTES-PDI-APTES@glass (tol, 12h)		12 h
APTES-PDI-APTES@glass (tol, 24h)		24 h
APTES-PDI-APTES@glass (tol, 48h)		48 h

### X-ray Photoelectron Spectroscopy

APTES-PDI-APTES@glass samples, prepared under various conditions, were first characterized by X-ray photoelectron spectroscopy (XPS) to gain insight into the chemical structure of the organic layer. Figure 3 presents a set of XPS spectra acquired for the layers deposited from toluene with an immersion time equal to 24 h. The survey spectrum recorded for APTES-PDI-APTES@glass (tol, 24h) (Figure 3A) reveals the presence of oxygen (O 1s peak at ca. 530 eV, O_KLL_ Auger peak at ca. 1000 eV), nitrogen (N 1s peak at ca. 400 eV), carbon (C 1s peak at ca. 285 eV), and silicon (Si 2p peak at ca. 100 eV and Si 2s at ca. 150 eV). The silicon and oxygen-related signals arise from both the glass substrate and layer and carbon C 1s signal from the organic layer and the adventitious carbon contaminations,^84–86^ while the nitrogen N 1s peak is specific to the layer. Analogous survey spectra were acquired for APTES-PDI-APTES@glass samples formed under different process conditions.

**Figure 3:**
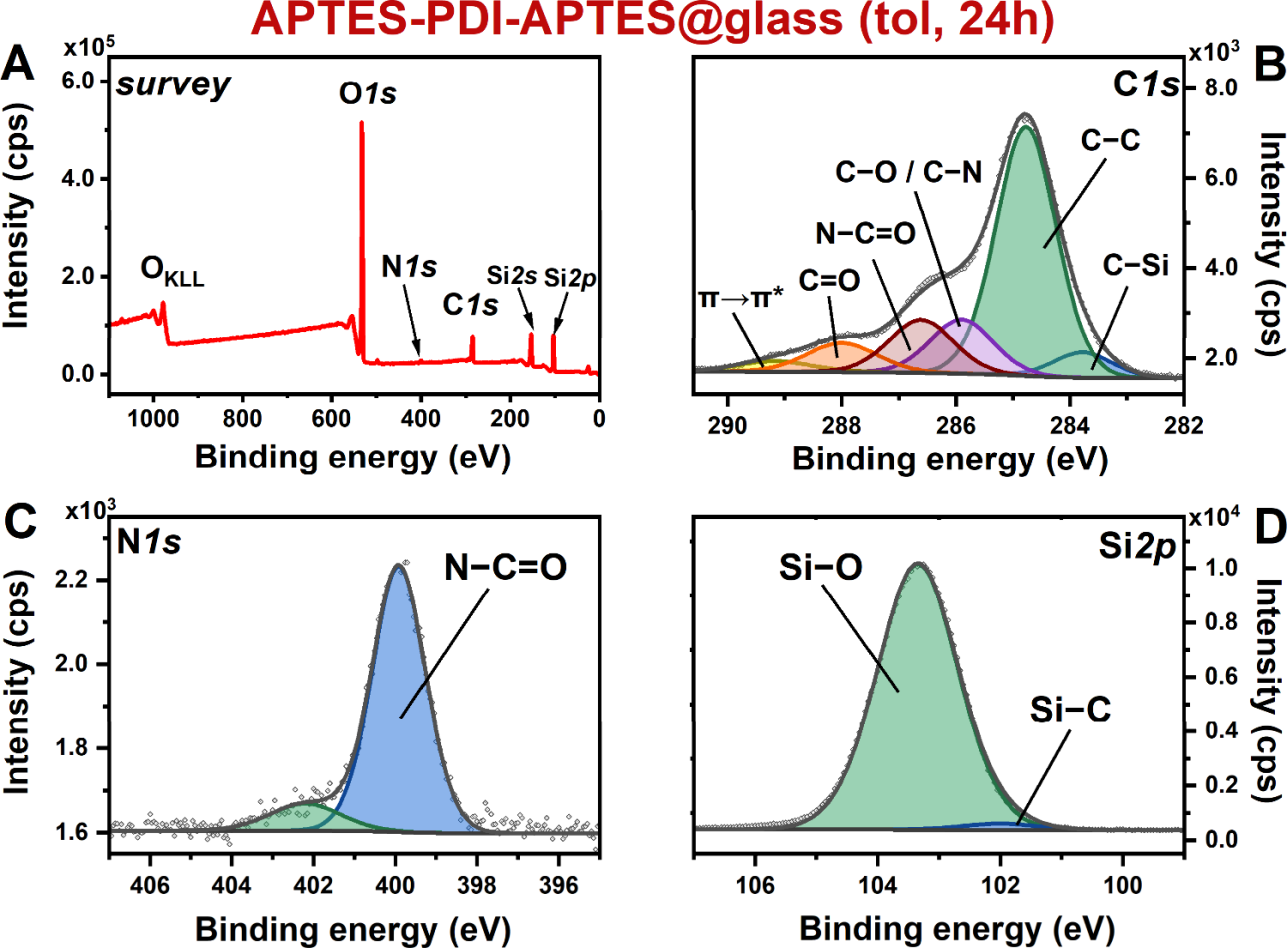
XPS (A) survey spectrum and high-resolution spectra of (B) C 1s, (C) N 1s, and (D) Si 2p regions recorded for sample APTES-PDI-APTES@glass (tol, 24h).

In the next step, the high-resolution spectra of the characteristic energy regions were analyzed. The decomposition of the C 1s high-resolution (HR) spectrum (Figure 3B, APTES-PDI-APTES@glass (tol, 24h)) gives six components located at 283.8, 284.8, 285.9, 286.6, 288.0, and 289.2 eV that can be assigned to C–Si, C–C, C–O/C–N, N–C=O, C=O, and π→π*, respectively.^84,87^ While all components can be assigned to the organic layer, C–C, C–O, and C=O can be also linked to adventitious carbon contaminations.^84^ Additionally, satellites from sp^2^ might also partially contribute to the C–O component.

The analysis of the HR N 1s spectrum (Figure 3C, APTES-PDI-APTES@glass (tol, 24h)) reveals the presence of two components at 400.1 and 402.2 eV. The first component can be assigned to nitrogen in PDI units,^88,89^ while the second one was unexpected. Importantly, when the N 1s HR spectrum was recorded at 45° (to make measurement more surface-sensitive, see Figure S4), the ratio between both components changes from 7 to 10, suggesting that the component at 402.2 eV arises from the units located closer to the glass surface. Finally, the Si 2p HR spectrum (Figure 3D) was fitted with two components: arising from the glass surface (SiO_2_ at 103.3 eV) and APTES units within the organic layer (Si–(OR)_3_ at 102.0 eV).^90,91^

The survey spectra and the high-resolution spectra of the specific energy regions recorded for APTES-PDI-APTES@glass samples deposited from DMF and DMSO showed similar features. The important difference in the acquired spectra lies in the intensity ratio of N 1s, C 1s, and Si 2p signals (Figure S5). The highest relative contribution of N 1s and C 1s signals was observed for toluene and the lowest for DMSO. This indicates that the surface grafting process is more efficient in the nonpolar solvents. Moreover, the analysis of N 1s HR spectra of the layers deposited from DMF and DMSO (Figure 4A,B) reveals the presence of two components (similarly to APTES-PDI-APTES@glass (tol, 24h)), but with an almost two times higher contribution of the component located at 402.2 eV. Thus, taking into account that an increase in solvent polarity leads to a higher relative intensity of the component at 402.2 eV and that it originates from regions closer to the glass surface (Figure S4), the component might be attributed to interactions between nitrogen atoms in the PDI units and hydroxyl (−OH) groups on the glass surface.^92–94^ This is further confirmed by the difference in the binding energies between N 1s components, which is in agreement with previous work^95^ reporting interactions between 3-aminopropyltrimethoxysilane and organosilicate surface. Those observations imply that some APTES-PDI-APTES molecules shall be flat on the glass surface to make such interactions possible. Still, it must be noted that the additional contribution to the component at 402.2 eV might also originate from the satellite from C–N in PDI. However, the arbitrary distinction is hard to assess.

**Figure 4:**
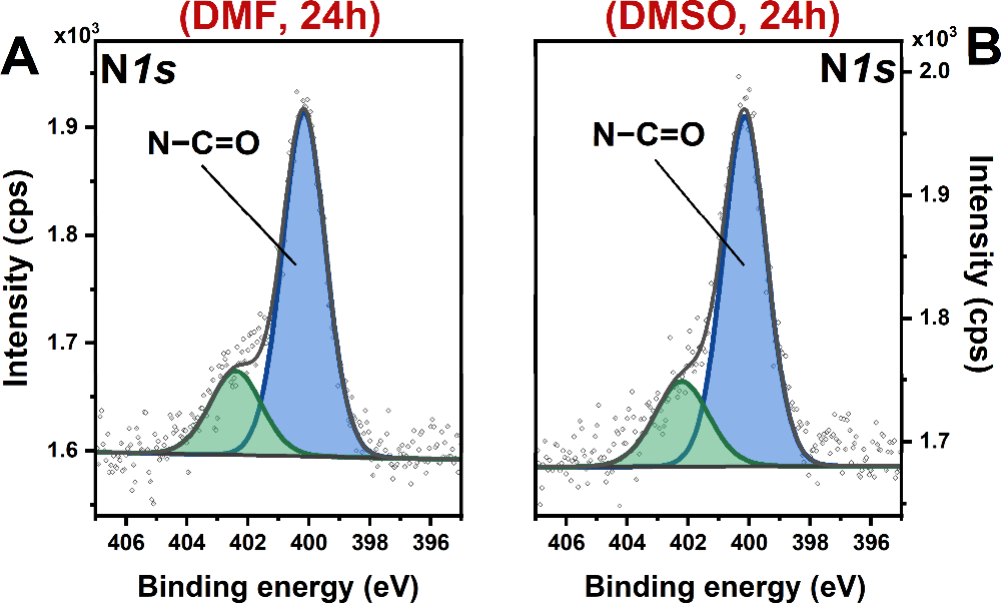
XPS high-resolution spectra of the N 1s region recorded for (A) APTES-PDI-APTES@glass (DMF, 24h) and (B) APTES-PDI-APTES@glass (DMSO, 24h).

Following the investigation of solvent effects on the structure of the deposited layer, the influence of the immersion time was studied. Toluene was chosen for these tests due to its highest surface grafting efficiency observed after 24 h. Figure S6 displays high-resolution N 1s spectra for APTES-PDI-APTES@glass samples prepared in toluene with varying deposition times. Consistent with the previously analyzed sample (tol, 24h), two distinct components were observed at 400.1 and 402.2 eV. Notably, the relative intensity of those components varied with immersion time (Table 3). While the differences between 6, 12, and 24 h are at the level of experimental uncertainty, the clear difference is observed between 3 and 48 h immersion times, suggesting the reconstruction and development of the APTES-PDI-APTES layer in time.^95^ Moreover, the relative contribution of C 1s signal changes from 22% for APTES-PDI-APTES@glass (tol, 3h) to 26% for APTES-PDI-APTES@glass (tol, 48h), which further indicates the growth of the organic layer.

**Table 3:** Contribution of N 1s Components for APTES-PDI-APTES@glass Deposited from Toluene with Varied Immersion Time

sample name	**% area of N** **1s** **components** **at 400.1 eV/at 402.2 eV** ^a^
APTES-PDI-APTES@glass (DMF, 24h)	78%/22%
APTES-PDI-APTES@glass (DMSO, 24h)	77%/23%
APTES-PDI-APTES@glass (tol, 3h)	82%/18%
APTES-PDI-APTES@glass (tol, 6h)	85%/15%
APTES-PDI-APTES@glass (tol, 12h)	86%/14%
APTES-PDI-APTES@glass (tol, 24h)	88%/12%
APTES-PDI-APTES@glass (tol, 48h)	90%/10%

a Experimental uncertainty ± 1%.

### Raman Spectroscopy

To further confirm the chemical structure of the deposited layer, Raman spectroscopy was employed. The Raman spectrum of the organic film formed on the glass surface within 24 h of immersion in APTES-PDI-APTES solution in toluene is shown in Figure 5. The vibration peaks characteristic of PDI units are observed in the range of 1300–1390 cm^–1^ and in the range of 1550–1600 cm^–1^, and they arise from C–H bending and C–C/C=C stretching in the perylene core, respectively.^96^ The characteristic vibration from APTES units, i.e., Si–C, was observed at ca. 1460 cm^–1^.^97,98^ Two other signals typically observed for alkoxysilanes, namely, Si–O stretching at ca. 800 cm^–^^1^ and Si–O–Si vibrations at ca. 1080 cm^–1^, overlap with the peaks originating from the glass substrate.^99^

**Figure 5:**
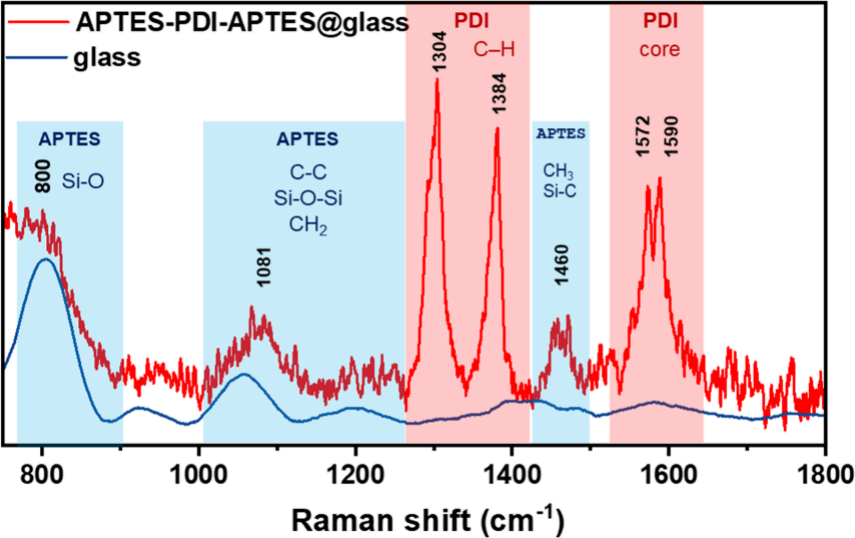
Raman spectra of APTES-PDI-APTES@glass (tol, 24h) (red line) and bare glass (blue line).

### Atomic Force Microscopy

The AFM analysis gave thorough insight into the layer formation process. Figure 6A–E presents consequent AFM images (top row) obtained for samples immersed in APTES-PDI-APTES toluene solution for 3–48 h. The images shown were chosen as a representative for each sample upon multiple scans in various places of the samples. However, it has to be underlined here that the entire surface of the sample was uniform regarding the topography. Further, in order to avoid possible image misinterpretation (also to ensure the extended level of objectivity), the obtained images underwent statistical analysis focused on roughness parameters and grain size distribution. Particularly, the latter is presented in the bottom row of Figure 6 in the form of mean grain size distribution histograms. The estimated roughness parameters, i.e., mean roughness (Sa) and root-mean-square of roughness (RMS) for each sample, are given in the right top corner of each respective histogram in the bottom row of Figure 6.

**Figure 6:**
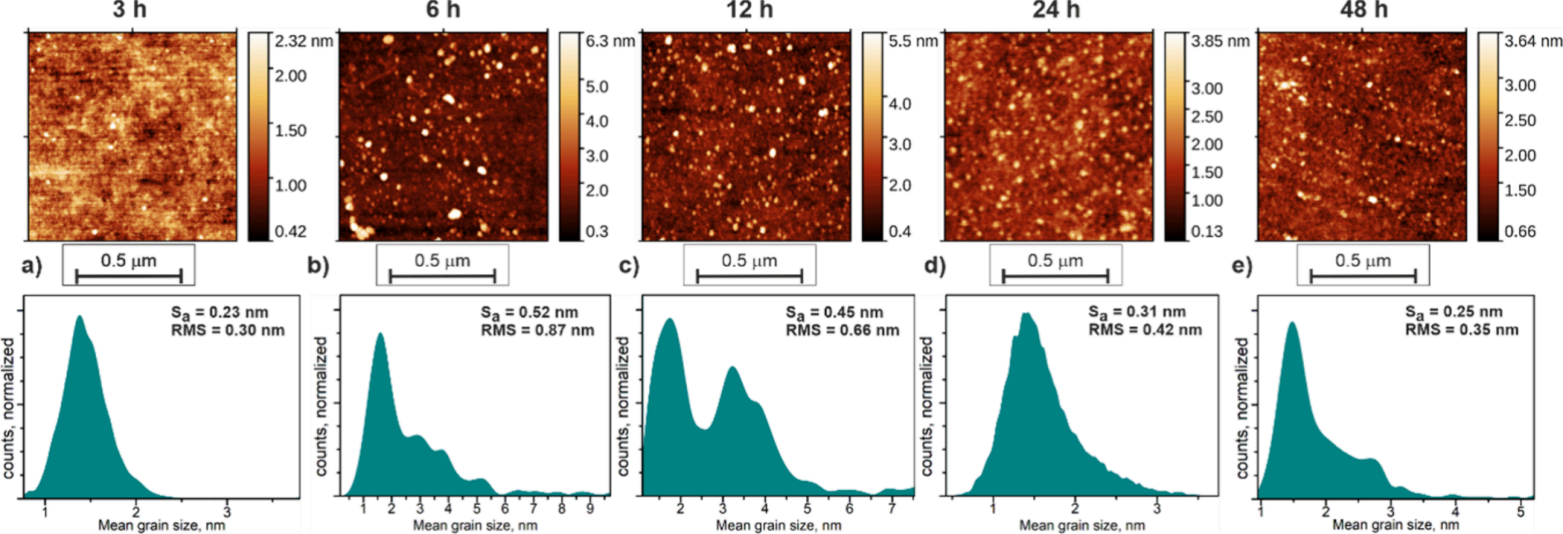
Exemplary results of AFM topography control obtained for APTES-PDI-APTES@glass (tol) with immersion times equal to 3 h (a), 6 h (b), 12 h (c), 24 h (d), and 48 h (e). The top row presents 1 × 1 μm^2^ topography images, while the bottom row depicts the results of statistical mean grain size distribution. The roughness parameters (i.e., mean roughness, *S*_a_, and root-mean-square of roughness, RMS) are given in the right top corner of each respective statistical panel.

Analyzing the topography images, the evolution of the surface as a function of time is clearly visible. One can see that after 3 h, the expected grainy structure is barely visible on the substrate surface. In fact, the surface is smooth at the level of the substrate with randomly appearing small nanograins. After 6 h, the grains are becoming significantly bigger with a strong tendency for protrusion from the substrate plane. The development of the surface (as well as the grain size) reaches its maximum after 12 h of immersion time. After that time, the tendency is most likely being reversed: after 24 h, the grains become smaller and the layer becomes smoother again. Finally after 48 h, the process results in obtaining a layer consisting of uniformly distributed small nanograins. The above consideration has strong support in mean grain size distribution results (see histograms in the bottom row of Figure 6). The mean grain size varies from 1.5 nm for the 3 h sample through ∼2 nm with a significant contribution in the range of 2–5 nm for the 6 h sample to the significantly disordered 12 h sample where the distribution has two maxima at ∼2 and 3.5 nm but with strong representation of grains in the whole 2–5 nm range. For this sample, larger crystallites up to 7 nm are also present in the statistical analysis. For the 24 and 48 h samples, one can observe the reversed tendency resulting in maxima in 1.5 nm with some contribution of grains in sizes up to 3 nm. A similar tendency can be observed for Sa and RMS parameters. However, in this case, the maximum roughness is detected for the 6 h sample. The important fact is that the algorithms used for mean grain size analysis and roughness analysis are completely independent of each other. Thanks to that, we can treat them as a kind of mutual cross-check minimizing the risk of faulty judgment.

As a conclusion from AFM analysis, we can propose the hypothesis that the layer aggregation occurs at ca. 6–12 h of deposition. In this time, the substrate–molecule interactions are smaller than those of the molecule–molecule, which results in the growth of grains together with their protrusion from the surface. This effect is often visible in deposition processes of large molecules like porphyrins or phthalocyanines.^100^ For a longer time, the reorganization of the layer occurs (most likely self-arrangement), which results in a more ordered surface with smaller crystallites. Smaller crystallites, in turn, make the surface smoother and more uniform.

### Singlet Oxygen Photogeneration

In the last step, the photoactivity of deposited layers was investigated applying a DPBF trap in methanol solution. Figure 7 presents the set of UV–vis spectra of DPBF recorded while in contact with the APTES-PDI-APTES@glass (tol, 48h) layer illuminated with a 532 nm diode laser. The decrease in the characteristic DPBF’s band at 410 nm indicates its oxidation with singlet oxygen produced by the layer. This proves that after immobilization, the PDI derivative retains its photoactivity toward ^1^O_2_ production. Notably, during the experiment, no new peak at ca. 500 nm appears, confirming that no PDI molecules go into the solution, and thus, singlet oxygen is produced by the layer.

**Figure 7:**
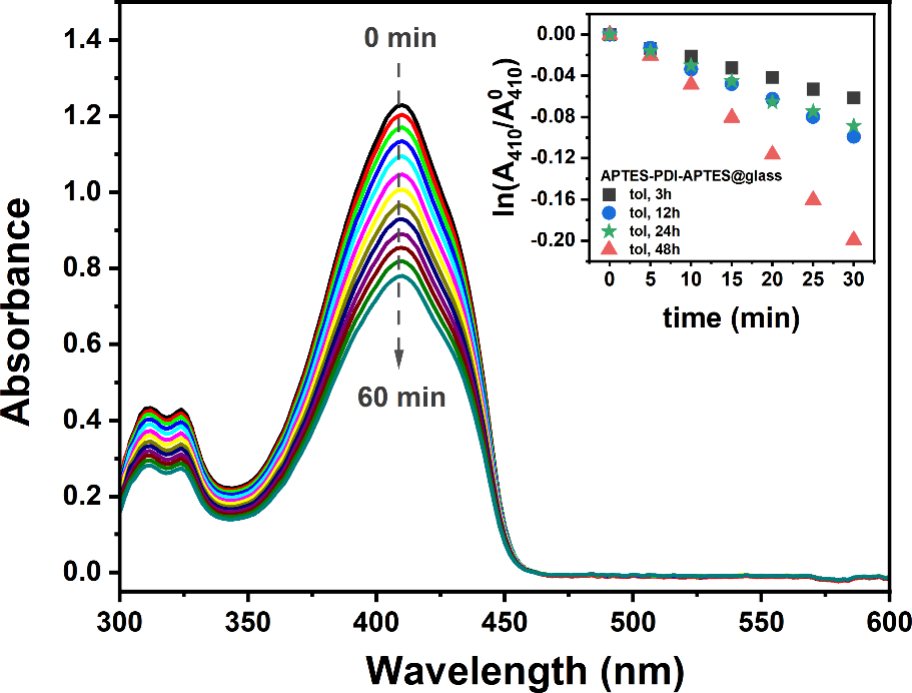
Set of UV–vis spectra of DPBF in methanol recorded during illumination of the APTES-PDI-APTES@glass (tol, 48h) layer with a 532 nm diode laser. The UV–vis spectra were recorded at time intervals equal to 5 min. Inset: drop in DPBF absorbance at 410 nm (given as a logarithmic function) in time observed during illumination of APTES-PDI-APTES@glass layers deposited from toluene solution with different immersion times.

Figure 7 (inset) presents the decrease in the absorbance of the above-mentioned band in time for various APTES-PDI-APTES@glass layers serving as a ^1^O_2_ source. The decrease in DPBF absorbance at 410 nm is presented as a logarithmic function, as its oxidation by singlet oxygen is considered a pseudo-first-order reaction. When the oxidation efficiency, used here as a measure of the photoactivity of the layer, is compared, it is generally observed that longer APTES-PDI-APTES deposition times result in higher rates of DPBF oxidation. This, of course, can be attributed to the increased amount of the photosensitizer immobilized on the glass surface. However, this correlation is slightly disturbed for 12 and 24 h deposition times, which exhibit comparable efficiencies. As observed by AFM, the APTES-PDI-APTES@glass (tol, 12h) layer is characterized by significantly higher RMS and Sa factors compared to the APTES-PDI-APTES@glass (tol, 24h) layer. Thus, the comparable photoactivity of those two layers is probably related to the significantly higher roughness of the APTES-PDI-APTES@glass (tol, 12h) layer, which enhances the heterogeneous process of ^1^O_2_ production. Those results prove that in the case of photoactive layers, both the amount of the deposited photosensitizer and morphology of the layer strongly influence the photoactivity of the system.

## 4 Conclusions

In this study, a perylene diimide (PDI) derivative bearing alkoxysilane groups (APTES-PDI-APTES) was synthesized and covalently grafted onto glass surfaces. The chemical composition and morphology of the resulting coatings were characterized by X-ray photoelectron spectroscopy, Raman spectroscopy, and atomic force microscopy. It was shown that the deposition parameters, i.e., solvent polarity and immersion time, strongly influence the structure and morphology of the formed layers. The lower polarity of a solvent enhances the immobilization of APTES-PDI-APTES on a surface, while prolonged immersion facilitates self-assembly into multilayer structures covalently bound to the glass surface. Importantly, the grafted PDI retains its photoactivity, as presented by singlet oxygen generation. This straightforward deposition approach based on chemical grafting can be possibly extended to other APTES-PDI derivatives, e.g., having electron-donating groups at the *bay* position, that would allow for the optimization of the layer absorbance and photoactivity. Finally, the described method may be used for the production of organic layers applicable as a singlet oxygen source, e.g., in light-activated antimicrobial coatings.

## Supplementary Material



## Data Availability

The data underlying this study are openly available in Zenodo.^101^

## Notes

Synthesis of *N*,*N*′-bis­[3-(triethoxysilyl)­propyl]­perylene-3,4,9,10-tetracarboxydiimide (APTES-PDI-APTES): synthetic procedure, ^1^H NMR spectrum (Figure S1), ^13^C NMR spectrum (Figure S2), HRMS data, and IR spectrum (Figure S3); X-ray photoelectron spectroscopy additional results: (Figure S4) XPS high-resolution spectrum of the N 1s region recorded for sample APTES-PDI-APTES@glass (tol, 24h) at a glancing angle of 45°, (Figure S5) XPS high-resolution spectra of the C 1s and Si 2p regions recorded for APTES-PDI-APTES@glass (DMF, 24h) and APTES-PDI-APTES@glass (DMSO, 24h), and (Figure S6) XPS high-resolution spectra of the N 1s region recorded for APTES-PDI-APTES@glass (tol, 3h), APTES-PDI-APTES@glass (tol, 6h), APTES-PDI-APTES@glass (tol, 12h), and APTES-PDI-APTES@glass (tol, 48h) (PDF)

## References

[1] Nuzzo R. G., Allara D. L. (1983). Adsorption of Bifunctional Organic Disulfides on Gold
Surfaces. J. Am. Chem. Soc..

[2] Seitz O., Böcking T., Salomon A., Gooding J. J., Cahen D. (2006). Importance
of Monolayer Quality for Interpreting Current Transport through Organic
Molecules: Alkyls on Oxide-Free Si. Langmuir.

[3] George S. M., Yoon B., Dameron A. A. (2009). Surface
Chemistry for Molecular Layer
Deposition of Organic and Hybrid Organic–Inorganic Polymers. Acc. Chem. Res..

[4] Casalini S., Bortolotti C. A., Leonardi F., Biscarini F. (2017). Self-Assembled
Monolayers in Organic Electronics. Chem. Soc.
Rev..

[5] Fukushima H., Seki S., Nishikawa T., Takiguchi H., Tamada K., Abe K., Colorado R., Graupe M., Shmakova O. E., Lee T. R. (2000). Microstructure, Wettability, and
Thermal Stability of Semifluorinated Self-Assembled Monolayers (SAMs)
on Gold. J. Phys. Chem. B.

[6] Sethuraman A., Han M., Kane R. S., Belfort G. (2004). Effect of Surface Wettability on
the Adhesion of Proteins. Langmuir.

[7] Zeng X., Xu G., Gao Y., An Y. (2011). Surface Wettability of (3-Aminopropyl)­Triethoxysilane
Self-Assembled Monolayers. J. Phys. Chem. B.

[8] Arima Y., Iwata H. (2007). Effect of Wettability
and Surface Functional Groups on Protein Adsorption
and Cell Adhesion Using Well-Defined Mixed Self-Assembled Monolayers. Biomaterials.

[9] Zorn G., Gotman I., Gutmanas E. Y., Adadi R., Salitra G., Sukenik C. N. (2005). Surface Modification
of Ti45Nb Alloy with an Alkylphosphonic
Acid Self-Assembled Monolayer. Chem. Mater..

[10] Ou J., Wang J., Liu S., Zhou J., Ren S., Yang S. (2009). Microtribological and
Electrochemical Corrosion Behaviors of Polydopamine
Coating on APTS-SAM Modified Si Substrate. Appl.
Surf. Sci..

[11] Chaki N. K., Vijayamohanan K. (2002). Self-Assembled Monolayers as a Tunable
Platform for
Biosensor Applications. Biosens. Bioelectron..

[12] Park B.-W., Kim D.-S., Yoon D.-Y. (2011). Surface Modification
of Gold Electrode
with Gold Nanoparticles and Mixed Self-Assembled Monolayers for Enzyme
Biosensors. Korean J. Chem. Eng..

[13] Choi S., Chae J. (2010). Methods of Reducing
Non-Specific Adsorption in Microfluidic Biosensors. J. Micromechanics Microengineering.

[14] Roh S., Jang Y., Yoo J., Seong H. (2023). Surface Modification
Strategies for Biomedical Applications: Enhancing Cell-Biomaterial
Interfaces and Biochip Performances. BioChip
J..

[15] Sánchez-Bodón J., Andrade Del Olmo J., Alonso J. M., Moreno-Benítez I., Vilas-Vilela J. L., Pérez-Álvarez L. (2022). Bioactive
Coatings on Titanium: A Review on Hydroxylation, Self-Assembled Monolayers
(SAMs) and Surface Modification Strategies. Polymers.

[16] Hasan A., Pandey L. M. (2015). Review: Polymers, Surface-Modified Polymers, and Self
Assembled Monolayers as Surface-Modifying Agents for Biomaterials. Polym.-Plast. Technol. Eng..

[17] Hau S. K., Cheng Y.-J., Yip H.-L., Zhang Y., Ma H., Jen A. K.-Y. (2010). Effect of Chemical Modification of Fullerene-Based
Self-Assembled Monolayers on the Performance of Inverted Polymer Solar
Cells. ACS Appl. Mater. Interfaces.

[18] Bedis H. (2011). Effect of
Self-Assembled Monolayers on the Performance of Organic Photovoltaic
Cells. J. Surf. Eng. Mater. Adv. Technol..

[19] Wen L., Gao F., Yu Y., Xu Z., Liu Z., Gao P., Zhang S., Li G. (2018). Enhancing the Photovoltaic Performance
of GaAs/Graphene Schottky Junction Solar Cells by Interfacial Modification
with Self Assembled Alkyl Thiol Monolayer. J.
Mater. Chem. A.

[20] Kim S. Y., Cho S. J., Byeon S. E., He X., Yoon H. J. (2020). Self-Assembled
Monolayers as Interface Engineering Nanomaterials in Perovskite Solar
Cells. Adv. Energy Mater..

[21] Börner, H. G. ; Lutz, J.-F. , Eds.; Bioactive Surfaces. Adv. Polym. Sci.; Springer Berlin Heidelberg: Berlin, Heidelberg, 2011; Vol. 240. 10.1007/978-3-642-20155-4.

[22] Lundin P. M., Fiser B. L., Blackledge M. S., Pickett H. L., Copeland A. L. (2022). Functionalized
Self-Assembled Monolayers: Versatile Strategies to Combat Bacterial
Biofilm Formation. Pharmaceutics.

[23] Sousa C., Teixeira P., Bordeira S., Fonseca J., R O. (2008). Staphylococcus
Epidermidis Adhesion to Modified Polycarbonate Surfaces: Gold and
SAMs Coated. J. Adhes. Sci. Technol..

[24] Zhang Z., Kou N., Ye W., Wang S., Lu J., Lu Y., Liu H., Wang X. (2021). Construction and Characterizations of Antibacterial
Surfaces Based on Self-Assembled Monolayer of Antimicrobial Peptides
(Pac-525) Derivatives on Gold. Coatings.

[25] McGovern M. E., Thompson M. (1998). Self-Assembled Silanes and the Thiol Functionalization
of Surfaces. Anal Commun..

[26] Haensch C., Hoeppener S., Schubert U. S. (2010). Chemical Modification of Self-Assembled
Silane Based Monolayers by Surface Reactions. Chem. Soc. Rev..

[27] Love J. C., Estroff L. A., Kriebel J. K., Nuzzo R. G., Whitesides G. M. (2005). Self-Assembled
Monolayers of Thiolates on Metals as a Form of Nanotechnology. Chem. Rev..

[28] Bürgi T. (2015). Properties
of the Gold-Sulphur Interface: From Self-Assembled Monolayers to Clusters. Nanoscale.

[29] Yu T., Marquez M. D., Tran H.-V., Lee T. R. (2022). Crosslinked Organosulfur-Based
Self-Assembled Monolayers: Formation and Applications. Soft Sci..

[30] Wang M., Liechti K. M., Wang Q., White J. M. (2005). Self-Assembled Silane
Monolayers: Fabrication with Nanoscale Uniformity. Langmuir.

[31] Wang Y., Lieberman M. (2003). Growth of
Ultrasmooth Octadecyltrichlorosilane Self-Assembled
Monolayers on SiO2. Langmuir.

[32] Paz Y. (2011). Self-Assembled
Monolayers and Titanium Dioxide: From Surface Patterning to Potential
Applications. Beilstein J. Nanotechnol..

[33] Grasset F., Saito N., Li D., Park D., Sakaguchi I., Ohashi N., Haneda H., Roisnel T., Mornet S., Duguet E. (2003). Surface Modification
of Zinc Oxide Nanoparticles by
Aminopropyltriethoxysilane. J. Alloys Compd..

[34] Allen C. G., Baker D. J., Albin J. M., Oertli H. E., Gillaspie D. T., Olson D. C., Furtak T. E., Collins R. T. (2008). Surface Modification
of ZnO Using Triethoxysilane-Based Molecules. Langmuir.

[35] Wang L., Schubert U. S., Hoeppener S. (2021). Surface Chemical Reactions on Self-Assembled
Silane Based Monolayers. Chem. Soc. Rev..

[36] Schwartz J., Avaltroni M. J., Danahy M. P., Silverman B. M., Hanson E. L., Schwarzbauer J. E., Midwood K. S., Gawalt E. S. (2003). Cell Attachment
and Spreading on Metal Implant Materials. Mater.
Sci. Eng., C.

[37] Rébiscoul D., Perrut V., Morel T., Jayet C., Cubitt R., Haumesser P.-H. (2010). Alkoxysilane Layers Compatible with
Copper Deposition
for Advanced Semiconductor Device Applications. Langmuir.

[38] Balasubramaniam B., Prateek, Ranjan S., Saraf M., Kar P., Singh S. P., Thakur V. K., Singh A., Gupta R. K. (2021). Antibacterial and Antiviral Functional
Materials: Chemistry and Biological Activity toward Tackling COVID-19-like
Pandemics. ACS Pharmacol. Transl. Sci..

[39] Socha K., Gusev I., Mroczko P., Blacha-Grzechnik A. (2025). Light-Activated
Antimicrobial Coatings: The Great Potential of Organic Photosensitizers. RSC Adv..

[40] Powell D., Whittaker-Brooks L. (2022). Concepts and
Principles of Self-n-Doping in Perylene
Diimide Chromophores for Applications in Biochemistry, Energy Harvesting,
Energy Storage, and Catalysis. Mater. Horiz.

[41] Peveler W.
J., Noimark S., Al-Azawi H., Hwang G. B., Crick C. R., Allan E., Edel J. B., Ivanov A. P., MacRobert A. J., Parkin I. P. (2018). Covalently Attached Antimicrobial Surfaces Using BODIPY:
Improving Efficiency and Effectiveness. ACS
Appl. Mater. Interfaces.

[42] Browne W. (2008). Photochemistry
of Immobilized Photoactive Compounds. Coord.
Chem. Rev..

[43] Condat M., Mazeran P.-E., Malval J.-P., Lalevée J., Morlet-Savary F., Renard E., Langlois V., Abbad Andalloussi S., Versace D.-L. (2015). Photoinduced Curcumin Derivative-Coatings with Antibacterial
Properties. RSC Adv..

[44] Savelyeva I. O., Zhdanova K. A., Gradova M. A., Gradov O. V., Bragina N. A. (2023). Cationic
Porphyrins as Antimicrobial and Antiviral Agents in Photodynamic Therapy. Curr. Issues Mol. Biol..

[45] Heredia D. A., Martínez S. R., Durantini A. M., Pérez M. E., Mangione M. I., Durantini J. E., Gervaldo M. A., Otero L. A., Durantini E. N. (2019). Antimicrobial
Photodynamic Polymeric Films Bearing
Biscarbazol Triphenylamine End-Capped Dendrimeric Zn­(II) Porphyrin. ACS Appl. Mater. Interfaces.

[46] Kim H.-S., Cha E. J., Kang H.-J., Park J.-H., Lee J., Park H.-D. (2019). Antibacterial Application
of Covalently Immobilized
Photosensitizers on a Surface. Environ. Res..

[47] Musolino S. F., Shatila F., Tieman G. M. O., Masarsky A. C., Thibodeau M. C., Wulff J. E., Buckley H. L. (2022). Light-Induced
Anti-Bacterial Effect
Against *Staphylococcus Aureus* of Porphyrin Covalently
Bonded to a Polyethylene Terephthalate Surface. ACS Omega.

[48] Ballatore M. B., Durantini J. E., Solis C., Boarini M. B., Gervaldo M., Otero L., Milanesio M. E., Durantini E. N. (2025). Photodynamic
Surfaces Coated with Porphyrin-Derived Polymers to Eradicate Staphylococcus
Aureus Biofilms. J. Photochem. Photobiol. Chem..

[49] Nyga A., Czerwińska-Główka D., Krzywiecki M., Przystaś W., Zabłocka-Godlewska E., Student S., Kwoka M., Data P., Blacha-Grzechnik A. (2021). Covalent Immobilization
of Organic Photosensitizers on the Glass Surface: Toward the Formation
of the Light-Activated Antimicrobial Nanocoating. Materials.

[50] López M., Gsponer N. S., Heredia D. A., Durantini E. N. (2025). Chlorin-Based
Photodynamic Antimicrobial Glass Surfaces for the Eradication of Staphylococcus
Aureus. Surf. Interfaces.

[51] Nzambe
Ta keki J. K., Ouk T. S., Zerrouki R., Faugeras P. A., Sol V., Brouillette F. (2016). Synthesis and Photobactericidal Properties of a Neutral
Porphyrin Grafted onto Lignocellulosic Fibers. Mater. Sci. Eng., C.

[52] Ringot C., Sol V., Barrière M., Saad N., Bressollier P., Granet R., Couleaud P., Frochot C., Krausz P. (2011). Triazinyl
Porphyrin-Based Photoactive Cotton Fabrics: Preparation, Characterization,
and Antibacterial Activity. Biomacromolecules.

[53] Li C., Wonneberger H. (2012). Perylene Imides
for Organic Photovoltaics: Yesterday,
Today, and Tomorrow. Adv. Mater..

[54] Kim S. H., Yang Y. S., Lee J. H., Lee J.-I., Chu H. Y., Lee H., Oh J., Do L.-M., Zyung T. (2003). Organic Field-Effect
Transistors Using Perylene. Opt. Mater..

[55] Li C., Gao Y., Huang R., Fang L., Sun Y., Yang Y., Gou S., Zhao J. (2022). An Effective Supramolecular
Approach to Boost the Photodynamic
Therapy Efficacy of a Near-Infrared Activating Perylene Diimide-Based
Photosensitizer. ACS Mater. Lett..

[56] Semeraro P., Syrgiannis Z., Bettini S., Giancane G., Guerra F., Fraix A., Bucci C., Sortino S., Prato M., Valli L. (2019). Singlet Oxygen
Photo-Production by Perylene Bisimide Derivative Langmuir-Schaefer
Films for Photodynamic Therapy Applications. J. Colloid Interface Sci..

[57] Özçil F., Yükrük F. (2023). Evaluation of Singlet
Oxygen Generators of Novel Water-Soluble
Perylene Diimide Photosensitizers. RSC Adv..

[58] Yukruk F., Dogan A. L., Canpinar H., Guc D., Akkaya E. U. (2005). Water-Soluble
Green Perylenediimide (PDI) Dyes as Potential Sensitizers for Photodynamic
Therapy. Org. Lett..

[59] Peres R. M., Brêda G. C., Almeida R. V., Corrêa R. J. (2021). Photochemistry
of Covalently Bonded Graphene Oxide – Perylene Diimide System
for Bacterial Growth Inhibition Started by Singlet Oxygen. J. Photochem. Photobiol. Chem..

[60] Dinçalp H., Kızılok Ş., İçli S. (2014). Targeted Singlet
Oxygen Generation Using Different DNA-Interacting Perylene Diimide
Type Photosensitizers. J. Fluoresc..

[61] Deckers J., Cardeynaels T., Lutsen L., Champagne B., Maes W. (2021). Heavy-Atom-Free Bay-Substituted
Perylene Diimide Donor-Acceptor Photosensitizers. ChemPhysChem.

[62] Rashed M. R., Sims C. B., Mahbub S., Hu N., Greene A. N., Espitia Armenta H., Iarussi R. A., Furgal J. C. (2024). Reinvigorating Photo-Activated
R-Alkoxysilanes Containing 2-Nitrobenzyl Protecting Groups as Stable
Precursors for Photo-Driven Si–O Bond Formation in Polymerization
and Surface Modification. ACS Omega.

[63] Castillo J. M., Klos M., Jacobs K., Horsch M., Hasse H. (2015). Characterization
of Alkylsilane Self-Assembled Monolayers by Molecular Simulation. Langmuir.

[64] Li P., Kangasniemi I., De Groot K., Kokubo T., Yli-Urpo A. U. (1994). Apatite
Crystallization from Metastable Calcium Phosphate Solution on Sol-Gel-Prepared
Silica. J. Non-Cryst. Solids.

[65] Wahab M. A., Hussain H., He C. (2009). Photoactive
Perylenediimide-Bridged
Silsesquioxane Functionalized Periodic Mesoporous Organosilica Thin
Films (PMO-SBA15): Synthesis, Self-Assembly, and Photoluminescent
and Enhanced Mechanical Properties. Langmuir.

[66] Su C., Hong B.-Y., Tseng C.-M. (2004). Sol-Gel
Preparation and Photocatalysis
of Titanium Dioxide. Catal. Today.

[67] Collinson M. M. (1999). Sol-Gel
Strategies for the Preparation of Selective Materials for Chemical
Analysis. Crit. Rev. Anal. Chem..

[68] Hwang I., Choi J., Rhee S.-H. (2023). Preparation
of a Thin Silica Nonwoven
Fabric Composed of Nanofibers and Micropores by Electrospinning after
Controlled Hydrolysis in a Sol-Gel Reaction. Ceram. Int..

[69] Dartar S., Ucuncu M., Karakus E., Hou Y., Zhao J., Emrullahoglu M. (2021). BODIPY–Vinyl Dibromides as
Triplet Sensitisers
for Photodynamic Therapy and Triplet-Triplet Annihilation Upconversion. Chem. Commun..

[70] Epelde-Elezcano N., Martínez-Martínez V., Peña-Cabrera E., Gómez-Durán C. F. A., Arbeloa I. L., Lacombe S. (2016). Modulation
of Singlet Oxygen Generation in Halogenated BODIPY Dyes by Substitution
at Their Meso Position: Towards a Solvent-Independent Standard in
the Vis Region. RSC Adv..

[71] Peddinti B. S. T., Morales-Gagnon N., Pourdeyhimi B., Scholle F., Spontak R. J., Ghiladi R. A. (2021). Photodynamic
Coatings
on Polymer Microfibers for Pathogen Inactivation: Effects of Application
Method and Composition. ACS Appl. Mater. Interfaces.

[72] Howarter J. A., Youngblood J. P. (2006). Optimization of Silica Silanization by 3-Aminopropyltriethoxysilane. Langmuir.

[73] Metwalli E., Haines D., Becker O., Conzone S., Pantano C. G. (2006). Surface
Characterizations of Mono-, Di-, and Tri-Aminosilane Treated Glass
Substrates. J. Colloid Interface Sci..

[74] Nečas D., Klapetek P. (2012). Gwyddion: An Open-Source Software for SPM Data Analysis. Open Phys..

[75] Data Levelling and Background Subtraction. http://gwyddion.net/documentation/user-guide-en/leveling-and-background.html (accessed 2025–06–04).

[76] Anguiano E., Aguilar M. (1999). A Cross-Measurement Procedure (CMP) for near Noise-Free
Imaging in Scanning Microscopes. Ultramicroscopy.

[77] Gwyddion. Scan Line Defects. Gwyddion Documentation. http://gwyddion.net/documentation/user-guide-en/scan-line-defects.html (accessed 2025–06–04).

[78] Gwyddion. Statistical Analysis. http://gwyddion.net/documentation/user-guide-en/statistical-analysis.html.

[79] Gwyddion. Grain Analysis. http://gwyddion.net/documentation/user-guide-en/grain-analysis.html.

[80] Bezboruah J., Mayurdhwaj Sanke D., Vinayakrao Munde A., Trilochand Bhattad P., Shekhar Karmakar H., Zade S. S. (2023). A TiO_2_ Nanorod and Perylene
Diimide Based Inorganic/Organic Nanoheterostructure Photoanode for
Photoelectrochemical Urea Oxidation. Nanoscale
Adv..

[81] Akash, Tiwari J. P. (2024). Synthesis and Characterization
of O-PDI for Futuristic Optoelectronic and Rectifier Applications. Mater. Adv..

[82] Howard J. A., Mendenhall G. D. (1975). Autoxidation
and Photooxidation of 1,3-Diphenylisobenzofuran:
A Kinetic and Product Study. Can. J. Chem..

[83] Brinker C.
J. (1988). Hydrolysis
and Condensation of Silicates: Effects on Structure. J. Non-Cryst. Solids.

[84] Beamson G., Briggs D. R. (1992). High Resolution
XPS of Organic Polymers: The Scienta
ESCA300 Database. J. Chem. Educ..

[85] Lößlein S.
M., Merz R., Müller D. W., Kopnarski M., Mücklich F. (2023). The Influence
of Adventitious Carbon Groups on the
Wetting of Copper: A Study on the Effect of Microstructure on the
Static Contact Angle. Langmuir.

[86] Grey L. H., Nie H.-Y., Biesinger M. C. (2024). Defining the Nature of Adventitious
Carbon and Improving Its Merit as a Charge Correction Reference for
XPS. Appl. Surf. Sci..

[87] Wang X.-L., Li X.-A., Tan M.-P., Liang Z.-Z., Chen Q.-Z., Huang J.-F., Xiao L.-M., Liu J.-M. (2024). Dye-Sensitized Photoelectrochemical
Cells Constructed Using Metal-Free Perylene Diimide-Based Oxygen Production
Polymers and Calixarene Dyes. J. Mater. Chem.
A.

[88] Russ B., Robb M. J., Popere B. C., Perry E. E., Mai C.-K., Fronk S. L., Patel S. N., Mates T. E., Bazan G. C., Urban J. J., Chabinyc M. L., Hawker C. J., Segalman R. A. (2016). Tethered
Tertiary Amines as Solid-State n-Type Dopants for Solution-Processable
Organic Semiconductors. Chem. Sci..

[89] Mazumder A., Sebastian E., Hariharan M. (2022). Solvent Dielectric Delimited Nitro-Nitrito
Photorearrangement in a Perylenediimide Derivative. Chem. Sci..

[90] Martin H. J., Schulz K. H., Bumgardner J. D., Walters K. B. (2007). XPS Study on the
Use of 3-Aminopropyltriethoxysilane to Bond Chitosan to a Titanium
Surface. Langmuir.

[91] Hafeez H., Choi D. K., Lee C. M., Jesuraj P. J., Kim D. H., Song A., Chung K. B., Song M., Ma J. F., Kim C.-S., Ryu S. Y. (2019). Replacement
of N-Type Layers with
a Non-Toxic APTES Interfacial Layer to Improve the Performance of
Amorphous Si Thin-Film Solar Cells. RSC Adv..

[92] Asenath
Smith E., Chen W. (2008). How To Prevent the Loss of Surface
Functionality Derived from Aminosilanes. Langmuir.

[93] Pasternack R. M., Rivillon
Amy S., Chabal Y. J. (2008). Attachment of 3-(Aminopropyl)­Triethoxysilane
on Silicon Oxide Surfaces: Dependence on Solution Temperature. Langmuir.

[94] Zarinwall A., Waniek T., Saadat R., Braun U., Sturm H., Garnweitner G. (2021). Comprehensive
Characterization of APTES Surface Modifications
of Hydrous Boehmite Nanoparticles. Langmuir.

[95] Fang J.-S., Yang T.-M., Pan Y.-C., Lai G.-Y., Cheng Y.-L., Chen G.-S. (2020). Chemical-Structure Evolution Model for the Self-Assembling
of Amine-Terminated Monolayers on Nanoporous Carbon-Doped Organosilicate
in Tightly Controlled Environments. Langmuir.

[96] Angelella M., Wang C., Tauber M. J. (2013). Resonance Raman Spectra of a Perylene
Bis­(Dicarboximide) Chromophore in Ground and Lowest Triplet States. J. Phys. Chem. A.

[97] Kerry R. G., Mohapatra P., Jena A. B., Panigrahi B., Pradhan K. C., Khatua B. R., Mahari S., Pal S., Perikala V., Kisan B., Lugos M. D., Mondru A. K., Sahoo S. K., Mandal D., Majhi S., Patra J. K. (2022). Biosynthesis
of Rutin Trihydrate Loaded Silica Nanoparticles and Investigation
of Its Antioxidant, Antidiabetic and Cytotoxic Potentials. J. Inorg. Organomet. Polym. Mater..

[98] Sun Y., Yanagisawa M., Kunimoto M., Nakamura M., Homma T. (2017). Depth Profiling
of APTES Self-Assembled Monolayers Using Surface-Enhanced Confocal
Raman Microspectroscopy. Spectrochim. Acta.
A. Mol. Biomol. Spectrosc..

[99] Glosz K., Ledwon P., Motyka R., Stolarczyk A., Gusev I., Blacha-Grzechnik A., Waskiewicz S., Kaluzynski P., Lapkowski M. (2022). Functionalized Polysiloxanes with
Perylene Diimides and Poly­(Ethylene Glycol): Synthesis and Properties. Eur. Polym. J..

[100] Niu T., Zhang J., Chen W. (2014). Molecular
Ordering and Dipole Alignment
of Vanadyl Phthalocyanine Monolayer on Metals: The Effects of Interfacial
Interactions. J. Phys. Chem. C.

[101] Socha, K. ; Krzywiecki, M. ; Mroczko, P. ; Nastula, D. ; Erfurt, K. ; Motyka, R. ; Blacha-Grzechnik, A. Insights into Growth of Photoactive Layer Based on Perylene Diimide Bearing Alkoxysilane Groups (Dataset) [Data set]. Zenodo., 2025 10.5281/ZENODO.16893131. PMC12490007 40976944

