# Supplementary material for: Insights into Growth of a Photoactive Layer Based on Perylene Diimide Bearing Alkoxysilane Groups
Source: Langmuir. 2025 Sep 22;41(38):26290–9. doi: 10.1021/acs.langmuir.5c03319 (PMC12490007; doi:10.1021/acs.langmuir.5c03319)
Supplement: Supplementary file 1 [file la5c03319_si_001.pdf]

Supporting Information

Insights into growth of photoactive layer based on perylene diimide bearing alkoxysilane groups

Karolina Socha [1], Maciej Krzywiecki [2], Patryk Mroczko [1], Dawid Nastula [1], Karol Erfurt [1], Radosław Motyka [3*], Agata Blacha-Grzechnik [1,4*]

[1] Faculty of Chemistry, Silesian University of Technology, Strzody 9, 44-100 Gliwice, Poland

[2] Institute of Physics – CSE, Silesian University of Technology, Konarskiego 22B, 44-100 Gliwice, Poland

[3] Centre of Polymer and Carbon Materials of the Polish Academy of Sciences, 34 M. Curie-Sklodowskiej St., 41-819 Zabrze, Poland

[4] Centre for Organic and Nanohybrid Electronics, Silesian University of Technology, 22B Konarskiego St., 44-100 Gliwice, Poland

* Corresponding authors:
ABG: agata.blacha@polsl.pl, tel. +48 322371024, Strzody 9, 44-100 Gliwice, Poland
RM: rmotyka@cmpw-pan.pl, tel. +48322716077 int.120, 34 M. Curie-Sklodowskiej St., 41-819 Zabrze, Poland

1. Synthesis of *N,N'-bis[3-(triethoxysilyl)propyl]perylene-3,4,9,10-tetracarboxdiimide* (APTES-PDI-APTES)

The synthesis of APTES-PDI-APTES was carried out according to the modified literature procedure[1] in a one-step reaction from perylene-3,4,9,10-tetracarboxylic dianhydride (0.78 g, 2.0 mmol) and (3-aminopropyl)triethoxysilane (3-APTES, 2.0 mL, 1.89 g, 8.6 mmol), as illustrated in ***Scheme S1***. The reagents were introduced into a single-neck round-bottom flask equipped with a magnetic stir bar and a reflux condenser, and the apparatus was thoroughly flushed with argon to ensure an inert atmosphere. The mixture was then heated to 130°C in an oil bath and stirred vigorously for 3 h, during which a homogeneous solution was obtained. After completion, the reaction mixture was allowed to cool to room temperature, and *n*-hexane (60 mL) was added, leading to precipitation of the desired product. The precipitate was isolated by filtration, washed repeatedly with *n*-hexane to remove excess 3-APTES, and subsequently dried under vacuum at room temperature. The target compound was obtained as a dark-red powder in 80% yield (1.28 g, 1.6 mmol).

^{1}H-NMR (400MHz, $CDCl_3$, ppm) (***Figure S1***): 8.58 (d, *J*=8.0Hz, 4H, $((O=)C)_2$-N-Ar-**H**), 8.46 (d, *J*=8.0Hz, 4H, Ar-**H**), 4.22-4.18 (m, 4H, N-C**H₂**), 3.84 (q, *J*=7.0Hz, 12H, O-C**H₂**), 1.93-1.85 (m, 4H, N-$CH_2C\mathbf{H_2}$), 1.23 (t, *J*=7.0Hz, 18H, $OCH_2C\mathbf{H_3}$), 0.80-0.76 (m, 4H, Si-$C\mathbf{H_2}$).

^{13}C-NMR (100MHz, $CDCl_3$, ppm) (***Figure S2***):163.3, 134.6, 131.4, 129.4, 126.5, 123.3, 123.0, 58.4, 43.1, 21.5, 18.3, 8.1.

HRMS (ESI-TOF) $[M+H]^+$ calcd. For $C_{42}H_{50}N_2O_{10}Si_2$ 799.3077, found 799.3038.

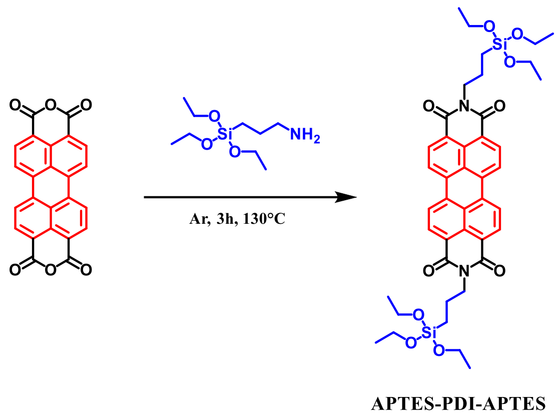

Scheme S1. *Scheme for the preparation of APTES-PDI-APTES*

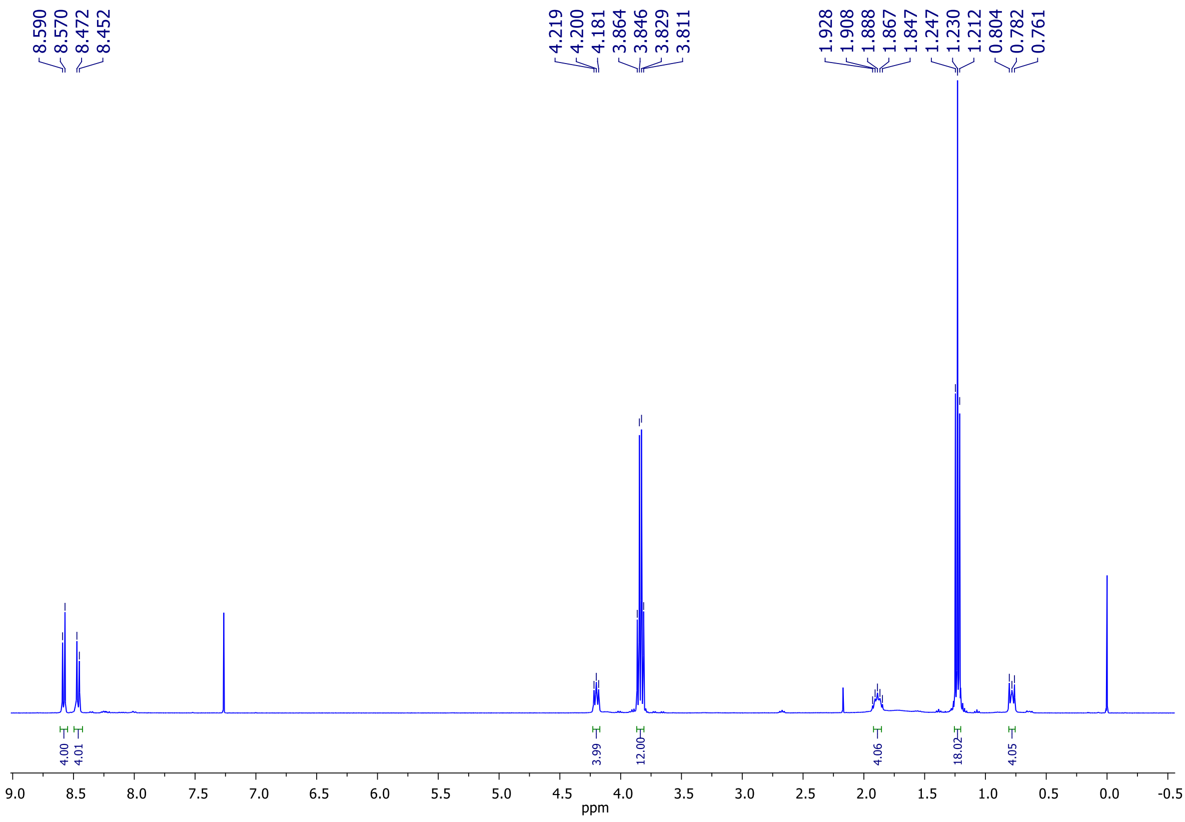

Figure S1. *^{1}H-NMR spectrum of APTES-PDI-APTES*

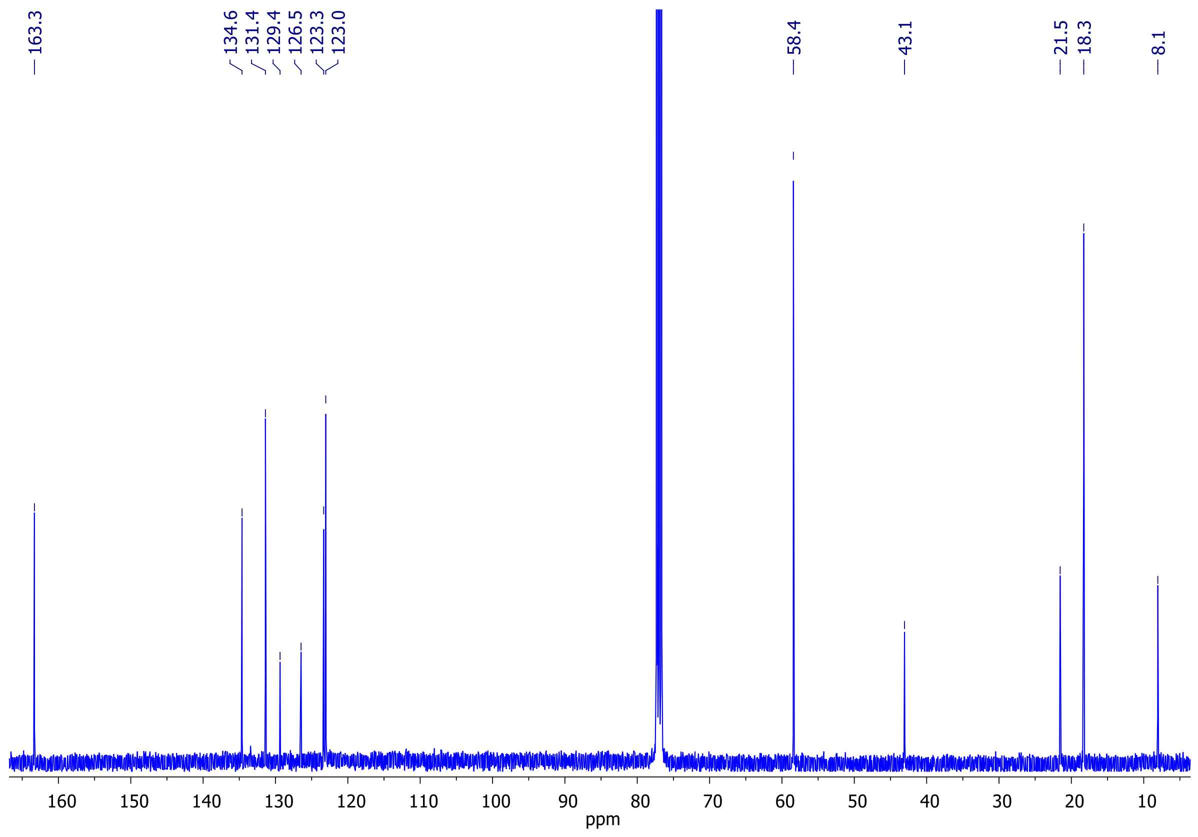

Figure S2. *^{13}C-NMR spectrum of APTES-PDI-APTES*

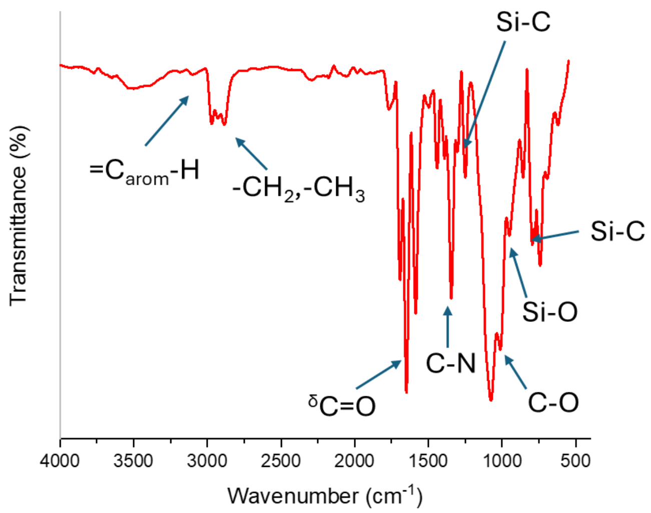

Figure S3. *FTIR spectrum of APTES-PDI-APTES*

Figure S3 presents the ATR-IR spectra recorded for APTES-PDI-APTES powder. IR spectrum showed the characteristic vibration bands of CH_2 and CH_3 framework at 2887 cm^{-1}, stretching vibrations of H-C bonds 2965 cm^{-1} and bending CH_2 and CH_3 at 1450 cm^{-1}. Next the presence of the C=O group was evident by stretching vibration at 1644 cm^{-1}.[2] Some of the most characteristic signals for APTES-PDI-APTES are the vibration bands of the Si-CH_2, Si-O bonds, which appeared on the spectrum at 1250 and 951 cm^{-1} respectively. [3,4] C-N stretching vibrations, originating from the formation of the imide part, and which can be observed at 1349 cm^{-1}.[5]

2. **X-ray Photoelectron spectroscopy of APTES-PDI-APTES@glass**

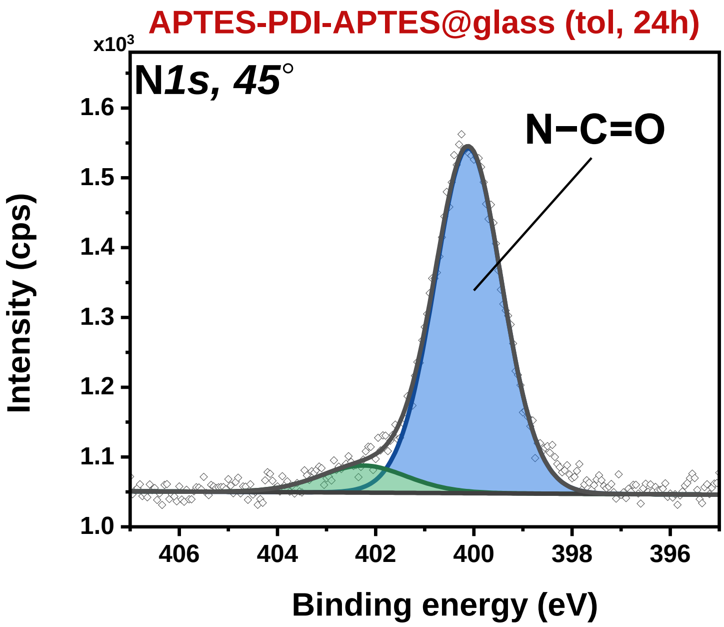

Figure S4. *XPS high-resolution spectrum of N 1s region recorded for APTES-PDI-APTES@glass (tol, 24h) at glancing angle 45°.*

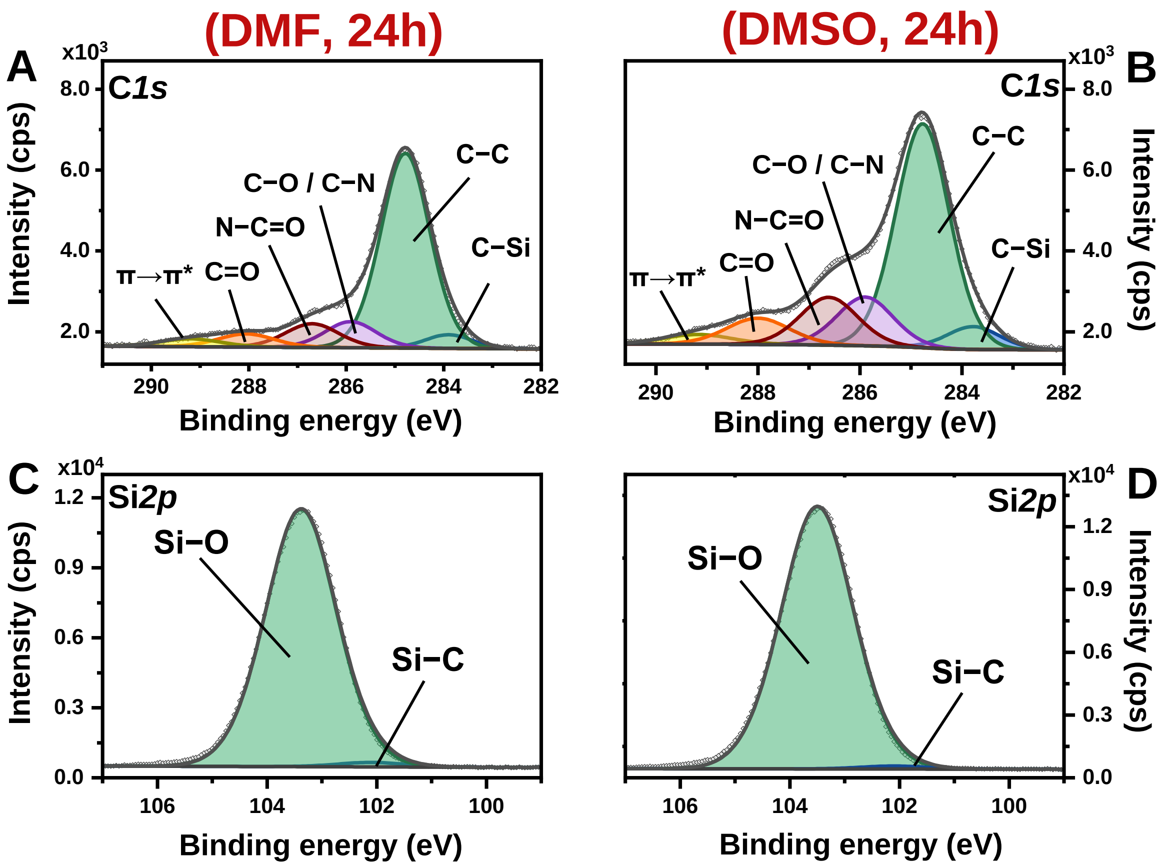

Figure S5. *XPS high-resolution spectra of C 1s and Si 2p regions recorded for A), C) APTES-PDI-APTES@glass (DMF, 24h) and B), D) APTES-PDI-APTES@glass (DMSO, 24h).*

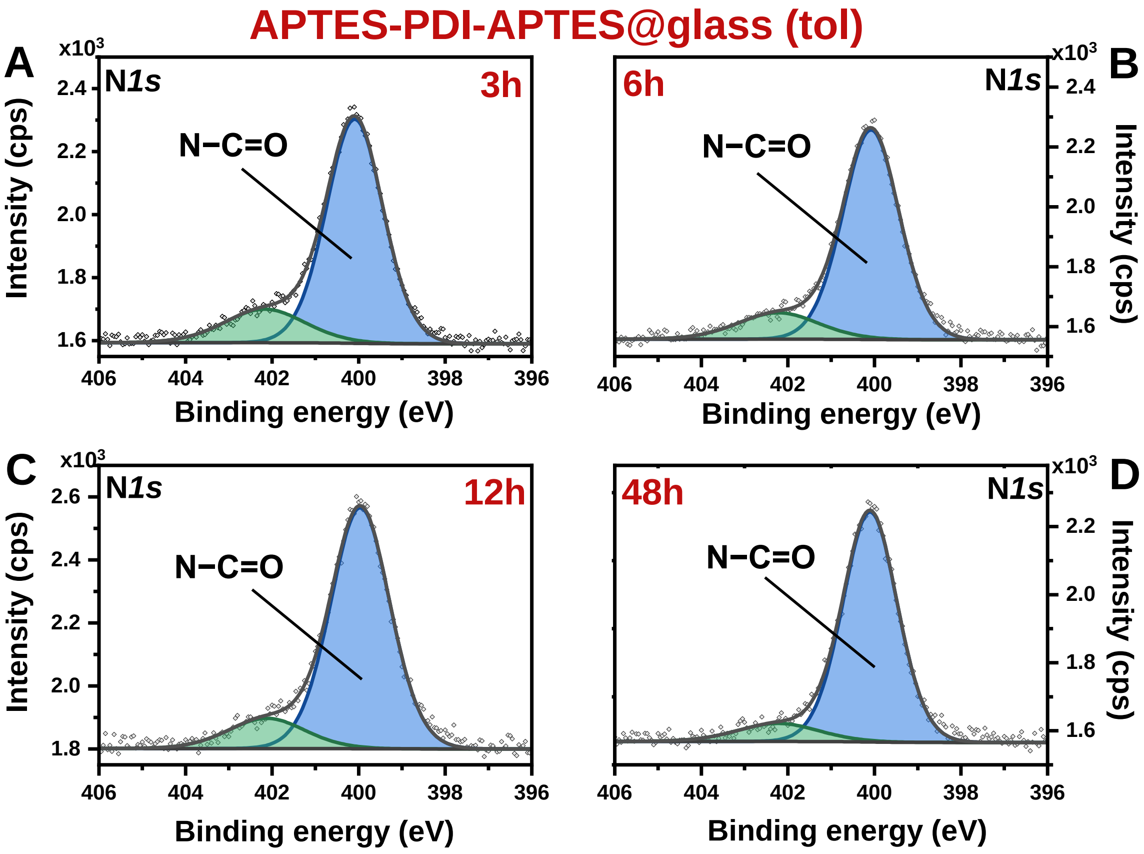

Figure S6. *XPS high-resolution spectra of N 1s region recorded for A) APTES-PDI-APTES@glass (tol, 3h), B) APTES-PDI-APTES@glass (tol, 6h), C) APTES-PDI-APTES@glass (tol, 12h,) and D) APTES-PDI-APTES@glass (tol, 48h).*

References:

(1) Wahab, M. A.; Hussain, H.; He, C. Photoactive Perylenediimide-Bridged Silsesquioxane Functionalized Periodic Mesoporous Organosilica Thin Films (PMO-SBA15): Synthesis, Self-Assembly, and Photoluminescent and Enhanced Mechanical Properties. *Langmuir* **2009**, *25* (8), 4743–4750. https://doi.org/10.1021/la900042g.

(2) Glosz, K.; Ledwon, P.; Motyka, R.; Stolarczyk, A.; Gusev, I.; Blacha-Grzechnik, A.; Waskiewicz, S.; Kaluzynski, P.; Lapkowski, M. Functionalized Polysiloxanes with Perylene Diimides and Poly(Ethylene Glycol): Synthesis and Properties. *Eur. Polym. J.* **2022**, *162*, 110878. https://doi.org/10.1016/j.eurpolymj.2021.110878.

(3) Chen, C.; Jia, Z.; Wang, X.; Lu, H.; Guan, Z.; Yang, C. Micro Characterization and Degradation Mechanism of Liquid Silicone Rubber Used for External Insulation. *IEEE Trans. Dielectr. Electr. Insul.* **2015**, *22* (1), 313–321. https://doi.org/10.1109/TDEI.2014.004188.

(4) Oh, T.; Choi, C. K. Comparison between SiOC Thin Film by Plasma Enhance Chemical Vapor Deposition and SiO2 Thin Film by Fourier Transform Infrared Spectroscopy. *J. Korean Phys. Soc.* **2010**, *56* (4), 1150–1155. https://doi.org/10.3938/jkps.56.1150.

(5) *Recent Trends in Materials Science and Applications: Nanomaterials, Crystal Growth, Thin Films, Quantum Dots, & Spectroscopy (Proceedings ICRTMSA 2016)*; Ebenezar, J., Ed.; Springer Proceedings in Physics; Springer International Publishing: Cham, 2017; Vol. 189. https://doi.org/10.1007/978-3-319-44890-9.
